# Association of Circulating miRNA Expression with Preeclampsia, Its Onset, and Severity

**DOI:** 10.3390/diagnostics11030476

**Published:** 2021-03-08

**Authors:** Zuzana Kolkova, Veronika Holubekova, Marian Grendar, Marcela Nachajova, Pavol Zubor, Terezia Pribulova, Dusan Loderer, Imrich Zigo, Kamil Biringer, Andrea Hornakova

**Affiliations:** 1 Biomedical Center Martin, Jessenius Faculty of Medicine in Martin, Comenius University in Bratislava, 036 01 Martin, Slovakia; veronika.holubekova@uniba.sk (V.H.); marian.grendar@uniba.sk (M.G.); dusan.loderer@uniba.sk (D.L.); a.mendelova@gmail.com (A.H.); 2 Department of Obstetrics and Gynecology, Jessenius Faculty of Medicine in Martin, Comenius University in Bratislava, Martin University Hospital, 036 01 Martin, Slovakia; marcela.nachajova@uniba.sk (M.N.); pribulova.terezia@gmail.com (T.P.); Imrich.Zigo@uniba.sk (I.Z.); kamil.biringer@uniba.sk (K.B.); 3 Department of Gynecologic Oncology, The Norwegian Radium Hospital, Oslo University Hospital, 0379 Oslo, Norway; prof.pavol.zubor@gmail.com; 4 OBGY Health & Care, Ltd., 010 01 Zilina, Slovakia

**Keywords:** miR-21-5p, miR-155-5p, miR-210-5p, miR-16-5p, preeclampsia, plasma, biomarkers

## Abstract

MicroRNAs (miRNAs) are one of the important regulators of cellular functions fundamental for healthy pregnancy processes, including angiogenesis and differentiation of trophoblast cells, and their deregulation could be implicated in the pathogenesis of pregnancy complications, including preeclampsia (PE). The aim of this study was to assess the association of miRNA expression in plasma samples with PE, its onset, and severity. Our study enrolled 59 pregnant women, 27 in the preeclamptic study group and 32 in the control group with physiological pregnancy. Preeclamptic pregnancies were divided into subgroups based on the severity and onset of disease. Relative expression of miR-21-5p, miR-155-5p, miR-210-5p, miR-16-5p, and miR-650 isolated from plasma samples was analysed by quantitative real-time PCR and normalised to experimentally established reference genes. Our results revealed upregulation of miR-21-5p (1.16-fold change, *p* = 0.0015), miR-155-5p (1.62-fold change, *p* = 0.0005) in preeclamptic pregnancies, compared to controls. Overexpression of these two miRNAs was observed, especially in subgroups of severe and late-onset PE compared to healthy pregnancies. Although we hypothesised that the expression level of studied miRNAs could vary between PE subtypes (mild vs. severe, early onset vs. late-onset), no obvious differences were detected. In conclusion, our study could contribute to the large-scale studies for the identification of non-invasive biomarkers for PE detection to improve outcomes for women and their new-borns.

## 1. Introduction

Preeclampsia (PE) is one of the most severe pregnancy complications that poses a risk to both mother and baby. This multisystem disorder affects approximately 5–8% of pregnancies, usually after the 20th gestational week, and is a leading cause of maternal and foetal mortality and morbidity [1]. PE is characterised mainly by high blood pressure (>140 mmHg systolic and/or >90 mmHg diastolic blood pressure) and proteinuria [2] and based on the American College of Obstetricians and Gynaecologists (ACOG) diagnostic criteria [3], PE can be classified as mild or severe. Early onset PE, occurring before the 33rd gestational week, represents a minority of cases but is associated with a higher risk of complications for the mother and her baby, compared to PE with late-onset [4,5]. The pathophysiology of PE remains unclear, but it is assumed that it has a multifactorial character and a critical role in its development plays an abnormal process of placentation and defects in placenta function, leading to reduced placental perfusion and hypoxia [6,7]. The development of the placenta involves various processes, such as proliferation, differentiation, invasion, angiogenesis, apoptosis, etc. [8,9]. All of these processes are modulated by physiological, genetic, environmental, and epigenetic factors, and their deregulation is associated with PE development and other pregnancy-related complications.

MicroRNAs (miRNAs) are single-stranded, 20–24 nt long, non-coding RNAs representing the epigenetic mechanism of posttranscriptional gene expression regulation. It is known that miRNAs act as regulators in various physiological processes, and their deregulation is one of the pathomechanisms of many diseases, including PE [10]. During pregnancy, miRNAs are expressed by placental tissue, and their concentration varies depending on the gestational week and stage of placental development, which highlights their importance in the regulation of placentation [9]. The placenta was found to express more than 500 miRNAs; many of them are characterised by specific expression patterns during pregnancy [11]. MicroRNAs of placental origin are released into circulation in the form of exosomes, microvesicles, apoptotic bodies, or bound to proteins [12], and might be detected in maternal serum or plasma. Since circulating miRNAs are relatively stable, and their concentration in plasma reflects expression patterns in the placenta, they are candidate non-invasive biomarkers for the detection of PE and other pregnancy-related complications [13]. Identification of specific and reliable biomarkers is a challenge, but it could improve diagnostics, management, and outcome of PE and decrease the risk of other complications associated with PE, mainly with the early onset and severe form.

The aim of our study was a comparison of expression profiles of selected miRNAs with a supposed role in PE pathogenesis between PE patients and physiological pregnancies. We also focused on the possible discrimination between mild and severe PE based on the expression of these miRNAs. Because there are differences in the pathogenesis of early and late-onset PE, we hypothesised that there could be differences in the expression pattern of selected miRNAs between these subtypes.

For this pilot study, we selected five miRNAs based on the literature review, namely, miR-210-5p, miR-155-5p, which are extensively studied in the context of PE; miR-21-5p and miR-16-5p with known expression in placenta, and also the little-studied miR-650. Based on current knowledge, these miRNAs are implicated in the regulation of cell processes important for proper placentation. Although these miRNAs were studied previously, study design and methodology were different among studies. In our pilot study, we decided to compare expression levels of selected miRNAs in maternal plasma collected just before delivery, similar to studies of placental tissue obtained after delivery. We believe that this study could be the basis for prospective studies monitoring differently expressed miRNAs during pregnancy, even before clinical manifestation of PE in suspected pregnancies that could be stratified based on other biochemical biomarkers.

## 2. Materials and Methods

### 2.1. Patients and Sampling

The plasma of patients with PE (*n* = 27) and normal pregnancy (*n* = 32), was collected from 1st January 2016 at the Department of Obstetrics and Gynaecology, Jessenius Faculty of Medicine and University Hospital Martin (Martin, Slovakia). The inclusion criteria for women with diagnosed PE was based on concomitant symptoms, such as high blood pressure as two or more entries of diastolic blood pressure of ≥90 mmHg taken ≥four hours apart and proteinuria as the secretion of ≥300 mg of protein over 24 h [14] after 20 weeks of gestation. The women with PE were divided into subgroups with early (before 34 weeks of pregnancy) and late-onset PE (after 34 weeks of pregnancy). The preeclamptic patients were also divided into subgroups based on the severity of PE. Severe PE was defined as severe gestational hypertension (systolic blood pressure ≥160 mmHg or diastolic ≥110 mmHg on two occasions at least four hours apart), based on ACOG criteria [3]. Foetal abdominal circumference (AC) or estimated foetal weight (EFW) < 10th centile was criterium for small for gestational age (SGA) [15]. Early foetal growth restriction (FGR) manifested before 32 weeks of gestational age was diagnosed based on criteria AC/EFW < 3rd centile or AC/EFW < 10th centile combined with uterine artery pulsatility index (UtA-PI) > 95th centile and/or umbilical artery pulsatility index (UA-PI) > 95th centile. Late FGR with manifestation ≥32 weeks of gestational age was defined based on AC/EFW < 3rd centile or at least two out of three parameters AC/EFW < 10th centile, AC/ EFW crossing centiles > 2 quartiles on growth centiles, CPR (cerebroplacental ratio) < 5th centile, or UA-PI > 95th centile [16,17].

Another set of healthy pregnant women belonged to the control group, with no pregnancy complications, such as artificial insemination, threatened abortion, premature rupture of membranes and/or premature birth, placenta praevia, and foetal macrosomia. All patients were Caucasians and agreed to be included in the cohort by signing informed consent. The study was conducted in accordance with the Declaration of Helsinki, and the protocol was approved by the Ethics Committee (no. EK-42/2018) at the Jessenius Faculty of Medicine, Comenius University in Bratislava, Martin, Slovakia.

A total of 10 mL of venous blood was taken from patients on admission to the hospital before delivery and processed within two hours after sampling. The blood was centrifuged in two steps to separate plasma at 3000 rpm for 10 min and pellet the cell debris at 14,000 rpm for 10 min. Plasma samples were stored at −80 °C prior to analysis. The presence of haemolysis in plasma samples was detected by measurement of oxyhaemoglobin absorbance at 414 nm. Only samples without apparent haemolysis were included in the study.

### 2.2. RNA Extraction and Reverse Transcription

Total RNA with miRNA fraction was extracted from 200 μL of plasma by miRNeasy Serum/Plasma Advanced kit (Qiagen, Hilden, Germany), according to manufacturer instructions. To control the isolation process, the mixture of synthetic spike-ins (RNA Spike-In Kit, Qiagen, Hilden, Germany) was added to the lysis buffer. RNA was dissolved in 20 μL of elution buffer and stored at −80 °C until the next step. One microliter of total RNA was reversely transcribed (miRCURY LNA RT kit, Qiagen, Hilden, Germany) into complementary DNA (cDNA) and stored at −20 °C.

### 2.3. miRNA Expression Analysis by Quantitative Real-Time PCR

One microliter of cDNA was diluted in 1:30 ratio, and three microliters of dilution were added into each real-time PCR reaction using miRCURY LNA miRNA PCR system (Qiagen, Hilden, Germany) with miRCURY LNA miRNA assays (Qiagen, Hilden, Germany) specific for reference (miR-103a-3p, miR-188-5p, miR-191-5p, and miR-222-3p) and target (miR-21-5p, miR-155-5p, miR-210-5p, miR-16-5p, and miR-650) miRNAs. Details of the miRNAs selected for this study are stated in Table 1. All 10 μL reactions were pipetted automatically on Bravo liquid handling station (Agilent Technologies, Santa Clara, CA, USA) to minimise subjective pipetting error. Quantitation of specific miRNAs with Cp determination by the second derivative method was run on LightCycler 480 (Roche Diagnostics GmbH, Mannheim, Germany) and related software.

### 2.4. Cp-Value Analysis

The raw analysis of Cp values was performed by the GeneGlobe data analysis tool, which is available at https://geneglobe.qiagen.com/us/ (accessed on 24 June 2020). The tool enables quality control of samples on the basis of spike-ins added during RNA extraction and reverse transcription, in addition to normalisation of data with a calculation of stability factor for reference miRNAs, according to the geNorm algorithm [18]. Samples with aberrant quality control were excluded from further analysis.

### 2.5. Statistical Analysis

Expression and fold change (FC) were computed using the standard formula [19]. The data were visualised by a boxplot overlaid with a swarm plot. The null hypothesis that the population median FC is equal to 1 was tested by the Wilcoxon test. The null hypothesis of the equality of the population median FC in the two populations (mild vs. severe) was tested by the Wilcoxon two-sample test (similarly for early and late-onset). Findings with a *p* value < 0.05 were considered statistically significant. The predictive ability of miRNA expression was assessed by the random forest (RF) machine learning algorithm. RF was trained on the data, and the nested cross-validation algorithm with the minimum graph depth criterion was used to select important features (miRNAs). The receiver operating characteristic (ROC) curve generated by the RF with selected miRNAs was used to quantify their predictive ability. The ROC curve was constructed from the out-of-bag data. The data analyses were performed in R [20] v. 3.5.2, using libraries beeswarm [21], robustbase [22], randomForestSRC [23], ggRandomForests [24].

## 3. Results

### 3.1. Characterisation of the Study Group

Our study enrolled 59 pregnant women aged 21–50 years old, of whom 27 were diagnosed with PE, and 32 women with physiological pregnancies were included in the control group. All pregnancies were singleton. Characteristics and basic clinical data of both groups are summarised in Table 2. As expected, the statistically significant differences between the groups were in parameters as systolic and diastolic blood pressure, pregnancy BMI, gestational age at delivery, and foetal birth weight. Foetal growth restriction was observed in eight PE pregnancies, of which two were early and six with late manifestation. Small for gestational age (SGA) status was observed in three PE cases. For the evaluation of the association between miRNA expression and PE severity and PE onset, 24 patients from the PE group were divided into subgroups. Overall, 13 patients with systolic blood pressure ≥160 mmHg or diastolic blood pressure ≥110 mmHg or both were classified as severe PE, and 11 patients with mild PE symptoms were included in another subgroup. The basic characteristics of both subgroups are listed in Table 3. Statistically significant differences between the subgroups were observed in blood pressure (systolic and diastolic). From the point of view of PE onset, patients were divided into two subgroups—early onset PE, occurring before the 33rd week of gestation (*n* = 7), and late-onset PE, which occurs after the 34th gestational week (*n* = 17). The characteristics of these subgroups are summarised in Table 4. As expected, there were statistically significant differences in gestational age at delivery and foetal birth weight.

Based on quality control of isolation and reverse transcription procedure through synthetic spike-ins, three samples from the control group were excluded from further miRNA expression analysis.

### 3.2. Selection of Reference Genes for Normalisation

Considering there are no miRNAs commonly used as endogenous reference genes, four candidate reference miRNAs were tested, namely miR-103a-3p, miR-188-5p, miR-191-5p, and miR-222-3p, for their stable expression across all our samples. The stability of expression was evaluated by using the geNorm algorithm. Our data were normalised to miR-103a-3p and miR-222-3p, which were identified as the most stably expressed genes across our samples in the control and study groups. The stability factor for miR-103-3p was 0.153, and for miR-222-3p, 0.094, which indicated low variability and high stability in expression between samples. The difference in average arithmetic mean for these two reference miRNAs was 0.12 cycles between cases and controls.

### 3.3. Different miRNA Expression in Plasma of PE Patients

**Figure 1:**
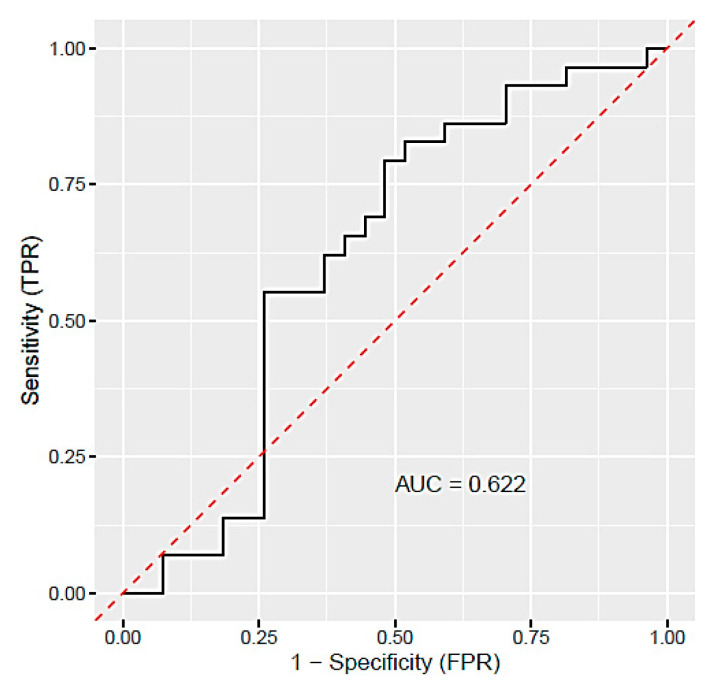
Receiver operating characteristic (ROC) curve analysis for the predictive potential of circulating miR-21-5p, miR-155-5p, and miR-16-5p (selected based on random forest analysis) in PE detection. The red line represents ROC curve of random classifier with AUC = 0.5; AUC, area under the curve.

Expression of five selected miRNAs (miR-16-5p, miR-21-5p, miR-155-5p, miR-210-5p, and miR-650) in plasma samples was analysed by qRT-PCR in 27 PE patients and 29 women with physiological pregnancies. Using this method, we detected a very poor expression of miR-650 in plasma samples, and therefore, we excluded this miRNA from further analysis. The relative expression of the remaining four miRNAs was computed by the delta–delta Ct method. Out of four studied miRNAs, miR-21-5p (1.16-fold change) and miR-155-5p (1.62-fold change) were upregulated with statistical significance (*p* < 0.05) in PE patients relative to controls (Table 5). To assess the predictive value of the studied miRNAs, the receiver operating characteristic (ROC) curve was generated. Based on random forest analysis, miR-21-5p, miR-155-5p, and miR-16-5p were selected as important predictors. According to the ROC curve (Figure 1) and area under the curve (AUC, 0.622), the predictive ability of these miRNAs is relatively low.

### 3.4. miRNA Expression in Context with Severity and Onset of Preeclampsia

**Figure 2:**
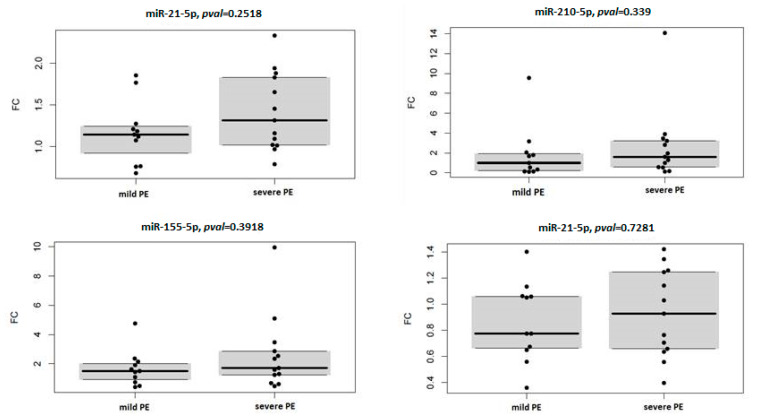
Comparison of miRNAs expression profiles between mild and severe PE subtypes. Abbreviation: FC, fold change relative to the control group.

**Figure 3:**
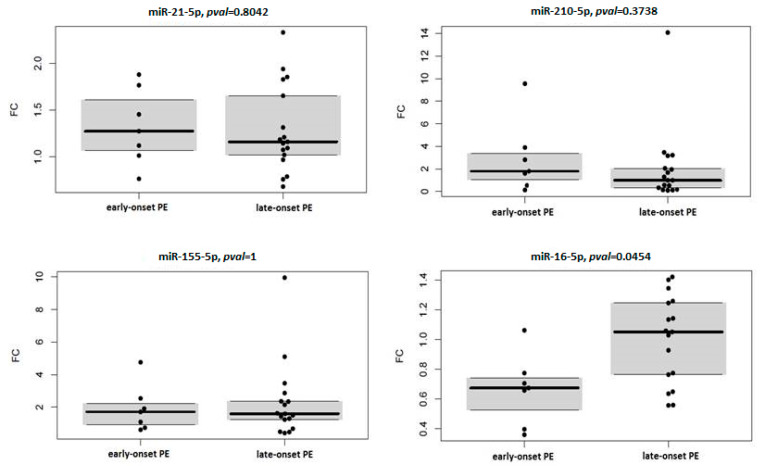
Comparison of miRNAs expression profiles between early and late-onset PE, Abbreviation: FC, fold change relative to the control group.

Fold change analysis of studied circulating miRNAs in context with severity and onset of PE showed overexpression of miR-21-5p and miR-155-5p in the case of severe PE (miR-21-5p: FC 1.316, *p* = 0.008; miR-155-5p: FC 1.711, *p* = 0.017) (Table 6) and late-onset PE (miR-21-5p: FC 1.162, *p* = 0.035; miR-155-5p: FC 1.597, *p* = 0.011) (Table 7) compared to healthy pregnancies. To assess the association of circulating miRNA expression with severity and onset of PE, expression profiles of four miRNAs relative to control samples were compared between subgroups of mild and severe PE, and early and late-onset PE, respectively. Results are visualised by box plots and swarm plots (Figure 2 and Figure 3) with respective *p* values. In the case of PE severity, no statistically significant differences were observed. In the case of PE onset, miR-16-5p was significantly under-expressed in the subgroup with early onset PE, compared to late-onset PE.

## 4. Discussion

Preeclampsia is classified as a complex, multisystemic, and multifactorial disease. Understanding its pathomechanisms is complicated, and the results of various studies are like pieces of the puzzle in this process. From a molecular point of view, deregulation of critical cellular processes, such as cell proliferation, migration, invasion, and apoptosis, is associated with improper placental development and function during pregnancy that leads to gestational complications. One of the molecular regulators of these processes is miRNAs expressed in the placenta. Their expression level is modulated by other factors, such as hypoxia, signalling pathways, or epigenetic modifications [25,26,27]. A recent meta-analysis confirmed the ability of circulating miRNAs to act as biomarkers of PE and indicated that their diagnostic value depends on specimen type, reference gene, and ethnicity [28]. Therefore, many other studies are necessary to confirm the predictive value and discrimination power of candidate miRNAs.

Based on the role of miRNAs in the pathogenesis of PE, miR-210 and miR-155 are the most extensively studied miRNAs in the context of PE biomarkers. Elevated levels of miR-210 have been detected in the placenta, and plasma of PE pregnancies in general [29,30,31,32,33,34,35], in addition to in placentas and maternal plasma of severe PE patients, compared to normal pregnancies [29,31,34,36,37,38]. Hypoxia, as a consequence of defective trophoblast invasion and spiral artery remodelling, is one of the factors inducing expression of miR-210 in the placenta, which leads to inhibition of trophoblast cell proliferation, invasion, and migration [30,39]. In our evaluation, we observed elevated expression of miR-210 in the PE group compared to controls and in the case of severe and early onset PE compared to healthy pregnancies, but the difference did not reach statistical significance. Based on our comparison of expression level between mild and severe PE and early and late-onset PE subtypes, expression of miR-210 did not vary significantly between these subgroups; hence, we can not assume that level of circulating miR-210 could distinguish PE subtypes from each other. The publication-based analysis provided by Tkachenko et al. (2020) showed that there could be several different mechanisms of PE development, according to the detection of dysregulated miRNAs, and not all of them are associated with miR-210 upregulation [40].

In preeclamptic conditions, the expression of miR-155 is induced by inflammatory factors [41], and its overexpression leads to the downregulation of target genes that are parts of pathways that are deregulated in preeclamptic pregnancy. Overexpression of circulating miR-155 in PE pregnancies was reported in several studies [42,43]. Murphy et al. (2015) showed a significant increase in miR-155 expression in the plasma of severe PE patients compared to controls, but not in the case of mild PE compared to controls [36], which is in agreement with the results of our study. In our retrospective study, we detected a 1.62-fold change with statistical significance in unselected PE patients compared to normal pregnancies. Our results also showed slight upregulation of miR-155 in severe PE and also in PE with late-onset compared to physiological pregnancies. On the other hand, we did not observe any significant differences in expression level when comparing PE patients divided based on the severity and onset of PE, respectively.

MiR-21 is an apoptosis-associated miRNA, and its expression was observed in the placenta, trophoblast, and even in maternal plasma [44,45,46]. The function of miR-21 in placenta development and PE pathogenesis is not entirely known, but it is proposed that it plays a role in the regulation of trophoblast cell function. Studies focused on the association between miR-21 expression and PE presented inconsistent results. Some studies reported overexpression of miR-21 in PE placentas [47,48], and also in maternal plasma or serum of PE patients [43,48]. Results of the study by Jairajpuri et al. (2017) depicted upregulation of circulating miR-21 in the plasma of patients with severe PE compared to the mild form [43]. In comparison, downregulation of miR-21 was observed in placentas affected by severe PE when compared to normal placentas [49] and also in plasma of PE patients, especially in the case of late-onset PE compared to healthy controls [50], which is contrary to our results that showed a slightly increased level of miR-21 expression (1.16-fold) in plasma of PE pregnancies compared to controls. Upregulated miR-21 was observed especially in subgroups of severe and late-onset PE compared to controls. However, we did not detect significant differences in its relative expression between early and late-onset PE or mild and severe forms of PE.

It has been shown that cell processes as proliferation [51,52], cell cycle [53,54], and angiogenesis [55,56] are regulated by miR-16, and it is assumed that aberrant expression of miR-16 could play a role in PE pathogenesis. The potential of miR-16 as a PE biomarker is questionable because some other studies did not detect any difference in miR-16 expression in the placenta [57] or plasma [50,58] of PE patients. On the other hand, the downregulation of miR-16 in plasma samples of women diagnosed with severe PE was described in the study of Wu et al. (2012) [59]. Our results revealed a slight decrease in the expression of circulating miR-16 in pregnancies with early onset PE compared to controls and also decreased level of expression in early onset PE compared to late-onset, in agreement with the findings of Dong et al. (2019) [50].

The function of miR-650 is not completely known. In previous studies, miR-650 was associated mainly with tumorigenesis [60,61,62]. The relationship between miR-650 and PE has not been widely studied. According to the study of Gunel et al. (2017), miR-650 is downregulated in placentas affected by severe PE [63]. On the other hand, in the case of circulating miR-650, its upregulation was reported in PE patients and also in severe PE form [38,43]. In our study, miR-650 was not detected in plasma samples by using a specific assay in qRT-PCR. The role of miR-650 in placental development, in addition to its deregulation in pregnancy complications, is questionable. Therefore, other studies focused on its targets and regulation pathways are necessary to uncover its biological functions.

In our study, miRNA expression was analysed by qRT-PCR, the most frequently used quantification method. Comparing the data obtained by this method between studies often leads to inconsistent results. This discrepancy could arise from the different sample sizes, study design, sample processing, procedures used, and the selection of reference genes used for normalisation that is critical for further data analysis [64]. There is a disadvantage that an established endogenous miRNA controls to normalise levels of miRNAs from plasma or serum are lacking [65]. In many studies, U6 and other small nuclear RNAs are widely used as reference genes even though they are more than 100nt long, which is much longer than target miRNAs and may result in different behaviour during isolation and reverse transcription [66]. Moreover, they are not suitable for studying miRNA expression in biofluids since they are not released and protected in circulation in the same way as miRNAs [67]. In general, it is highly recommended to determine stably expressed miRNA experimentally for each study and to use a combination of two to three reference genes for normalisation [18,66,68]. Of the four potential reference genes tested in our study (miR-103a-3p, miR-188-5p, miR-191-5p, and miR-222-3p), the combination of miR-103a-3p and miR-222-3p proved to be the most stably expressed and suitable for normalisation based on the geNorm algorithm. We consider this approach of experimentally established endogenous controls together with quality control of isolation and reverse transcription procedure by exogenous spike-in controls to be the strength of our study. In contrast, the limitation of our and other studies is a relatively small study group. In the future, it is necessary to standardise normalisation methods and interpretation of results to overcome discrepancies between studies and to obtain reliable and robust results. In addition, to assess the discrimination power and diagnostic value of miRNAs, it is important to validate the results in a large number of patients.

The results of our pilot study reflect the association of circulating miRNAs with PE and its subtypes after PE onset, in time before delivery. This study design is similar to studies of miRNA expression in the placenta which are obtained after delivery. By this strategy, we are not able to distinguish whether aberrant miRNA expression detected in the plasma of PE pregnancies contributes to PE development or it is a consequence of pathological processes and endothelial damage. However, observed differences can indicate the potential of miR-155-5p and miR-21-5p in the detection of PE, especially severe and late-onset form. To confirm this assumption, it is necessary to study their expression profiles in different stages of PE pregnancy in comparison with gestational week-matched healthy pregnancies. When considering these miRNAs as early biomarkers, it would be appropriate to focus future studies on the detection of their expression level in the preclinical stages of PE. Unfortunately, this is very complicated as PE is diagnosed after clinical manifestation based on gold standard clinical, ultrasound, and laboratory criteria. Diagnostics of PE can be improved also by biochemical biomarkers detectable in serum.

Based on three large-scale prospective studies, screening of maternal factors, uterine artery pulsatility index (UtA-PI), mean arterial pressure (MAP) and placental growth factor (PlGF) at 11–13 gestational week is the best screening approach for prediction of PE, especially early PE, in the first trimester so far [69,70,71]. Diagnostics of PE in the second and third trimester may be improved by measurement of angiogenic factors, mainly sFlt-1/PlGF ratio (soluble fms-like tyrosine kinase-1/placental growth factor). According to studies, the sFlt-1/PlGF ratio has high negative but quite low positive predictive value [72,73,74]; therefore, it is recommended mainly for ruling out PE in pregnancies with suspected PE [75,76,77]. There are also other serum biomarkers as soluble endoglin (sEng), placental protein 13 (PP13), pregnancy-associated plasma protein-A (PAPP-A), etc., but their sensitivity and specificity alone or combined with other biomarkers need to be confirmed in large-scale studies. The current knowledge of biochemical biomarkers could be useful also for the stratification of suspected PE pregnancies for future prospective studies of circulating miRNA expression to assess their predictive value and ability to become reliable non-invasive biomarkers.

In conclusion, our study of miRNA expression profiles in PE and its subtypes revealed the slight upregulation of circulating miR-155-5p and miR-21-5p. Our predictive model involving miR-155-5p, miR-21-5p, and miR-16-5p as predictors of PE showed poor discrimination power. The mild differences could be due to relatively small study groups. Therefore, more studies with a greater sample size will be necessary to assess the diagnostic potential of these miRNAs. Although we hypothesised that the expression level of studied miRNAs could vary between PE subtypes (mild vs. severe, early onset vs. late-onset), no statistically significant results were observed. Mild overexpression of miR-155-5p and miR-21-5p was observed in the case of a severe form of PE and PE with late-onset. Moreover, mild downregulation of miR-16-5p was associated with early onset PE. We did not detect the expression of miR-650 in plasma samples.

In our future study of the association between miRNA expression and PE, we plan to implement an NGS (next generation sequencing) methodology that provides a sensitive and complex approach for absolute miRNA quantification, and detection of novel miRNAs. We hope that our current study and our future studies will provide pieces of the puzzle in search of reliable biomarkers for PE detection to improve outcomes for women and their new-borns.

## Figures and Tables

**Table 1:** Characteristics of microRNAs included in the study.

miRBase ID	Assay Catalog Number	Coordinates (GRCh38)	miRNA Sequence
hsa-miR-16-5p	YP00205702	chr13: 50048973-50049061 [−]	5‘-UAGCAGCACGUAAAUAUUGGCG-3‘
hsa-miR-21-5p	YP00204230	chr17: 59841266-59841337 [+]	5‘-UAGCUUAUCAGACUGAUGUUGA-3‘
hsa-miR-103a-3p	YP00204063	chr20: 3917494-3917571 [+]	5‘-AGCAGCAUUGUACAGGGCUAUGA-3‘
hsa-miR-155-5p	YP00204308	chr21: 25573980-25574044 [+]	5‘-UUAAUGCUAAUCGUGAUAGGGGUU-3‘
hsa-miR-188-5p	YP00204239	chrX: 50003503-50003588 [+]	5‘-CAUCCCUUGCAUGGUGGAGGG-3‘
hsa-miR-191-5p	YP00204306	chr3: 49020618-49020709 [−]	5‘-CAACGGAAUCCCAAAAGCAGCUG-3‘
hsa-miR-210-5p	YP00204321	chr11: 568089-568198 [−]	5‘-AGCCCCUGCCCACCGCACACUG-3‘
hsa-miR-222-3p	YP00204551	chrX: 45747015-45747124 [−]	5‘-AGCUACAUCUGGCUACUGGGU-3‘
hsa-miR-650	YP00204233	chr22: 22822776-22822871 [+]	5‘-AGGAGGCAGCGCUCUCAGGAC-3‘

**Table 2:** Clinical characteristics of physiological and preeclamptic pregnancies.

Parameter	Healthy Pregnant Women	PE Patients	*p* Value
	(*n* = 32)	(*n* = 27)	
Age (years)	30 (25–37)	27 (21–50)	0.072
Blood pressure (mmHg)			
Systolic	128 (99–147)	157 (125–198)	<0.001
Diastolic	79 (54–91)	102.5 (75–120)	< 0.001
Proteinuria (g/24 h)	None	1.54 (0.126–9.86)	-
Pregnancy body mass index	26.5 (20.4–40.4)	30.8 (20.2–44.6)	<0.001
Gestational age at delivery (weeks)	40 (37–42)	37.5 (27–42)	<0.001
Mode of delivery			0.01
Vaginal	20 (62.5%)	9 (33.3%)	
Caesarean section	9 (28.1%)	15 (55.5%)	
NA	3 (9.4%)	3 (11.1%)	
Foetal birth weight (grams)	3535 (2630–4540)	2800 (590–4290)	0.002
Foetal growth restriction early late SGA Foetal sex	− − − −	8 (29.6%) 2 (7.4%) 6 (22.2%) 3 (11.1%)	
Boy	14 (43.75%)	9 (33.3%)	
Girl	15 (46.88%)	14 (51.9%)	
NA	3 (9.4%)	4 (14.8%)	

Median with corresponding minimum and maximum in brackets or a number and percent in brackets are present for each variable. Abbreviation: NA, not available; SGA, small for gestational age.

**Table 3:** Clinical characteristics of preeclamptic pregnancies divided into subgroups based on the severity of preeclampsia (PE).

Parameter	Patients with Mild PE	Patients with Severe PE	*p* Value
	(*n* = 11)	(*n* = 13)	
Age (years)	24.5 (21–32)	29 (22–50)	0.051
Blood pressure (mmHg)			
Systolic	144 (125–160)	164 (134–198)	0.002
Diastolic	96 (75–106)	106 (84–120)	0.02
Proteinuria (g/24 h)	1.41 (0.297–3.66)	1.8 (0.126–9.86)	0.7
Pregnancy body mass index	29.1 (20.2–44.6)	31.5 (24.6–41.6)	0.3
Gestational age at delivery (weeks)	38 (27–42)	37 (28–41)	0.8
Mode of delivery			0.2
Vaginal	6 (54.5%)	3 (23.1%)	
Caesarean section	5 (45.5%)	10 (76.9%)	
Foetal birth weight (grams)	2440 (590–3900)	2930 (660–4290)	0.6
Foetal sex			0.3
Boy	5 (45.5%)	9 (69.2%)	
Girl	6 (54.5%)	3 (23.1%)	
NA	0	1 (7.7%)	

Median with the corresponding minimum and maximum in brackets or a number and percent in brackets are present for each variable; Abbreviation: NA, not available.

**Table 4:** Clinical characteristics of preeclamptic pregnancies divided into subgroups based on onset of PE.

Parameter	Patients with Early Onset PE	Patients with Late-Onset PE	*p* Value
	(*n* = 7)	(*n* = 17)	
Age (years)	27 (21–50)	26.5 (22–34)	>0.9
Blood pressure (mmHg)			
Systolic	155 (143–189)	157 (125–180)	0.6
Diastolic	102 (81–110)	104 (75–116)	0.5
Proteinuria (g/24 h)	1.8 (1.286–3.66)	1.04 (0.126–9.86)	0.3
Pregnancy body mass index	28.4 (20.2–44.6)	31.1 (23.4–44.6)	0.6
Gestational age at delivery (weeks)	31 (27–34)	39 (35–41)	<0.001
Mode of delivery			0.01
Vaginal	0 (0%)	9 (52.9%)	
Caesarean section	7 (100%)	8 (47.1%)	
Foetal birth weight (grams)	1460 (590–1790)	3140 (1600–4290)	<0.001
Foetal sex			>0.9
Boy	4 (57.1%)	10 (58.8%)	
Girl	2 (28.6%)	7 (41.2%)	
NA	1 (14.3%)		

Median with corresponding minimum and maximum in brackets or a number and percent in brackets are present for each variable. Abbreviation: NA, not available.

**Table 5:** Fold change analysis of studied miRNAs detected in plasma samples of PE patients compared to healthy pregnancies.

miRNA	Median Fold Change	*p* Value
hsa-miR-16-5p	0.78	0.0903
hsa-miR-21-5p	1.16	0.0015
hsa-miR-155-5p	1.62	0.0005
hsa-miR-210-5p	1.29	0.0735

Fold change > 1 indicates upregulation, whereas < 1 indicates downregulation.

**Table 6:** Relative expression of circulating miRNAs in patients with mild and severe PE.

miRNA	Mild PE		Severe PE	
	Median FC	*p* Value	Median FC	*p* Value
hsa-miR-16-5p	0.776	0.23	0.929	0.497
hsa-miR-21-5p	1.146	0.365	1.316	0.008
hsa-miR-155-5p	1.505	0.083	1.711	0.017
hsa-miR-210-5p	0.991	0.638	1.604	0.094

PE, preeclampsia; FC, fold change.

**Table 7:** Relative expression of circulating miRNAs in patients with early and late-onset PE.

miRNA	Early Onset PE		Late-Onset PE	
	Median FC	*p* Value	Median FC	*p* Value
hsa-miR-16-5p	0.675	0.031	1.052	1
hsa-miR-21-5p	1.275	0.078	1.162	0.035
hsa-miR-155-5p	1.711	0.156	1.597	0.011
hsa-miR-210-5p	1.792	0.156	0.991	0.344

PE, preeclampsia; FC, fold change.

## Data Availability

The datasets that support the presented results of this study are available on reasonable request from the corresponding author.
